# MR flow measurement of coronary artery bypass graft and stress myocardial perfusion MRI for the detection of graft stenosis

**DOI:** 10.1186/1532-429X-15-S1-P178

**Published:** 2013-01-30

**Authors:** Tatsuro Ito, Masaki Ishida, Kakuya Kitagawa, Hiroshi Nakajima, Kaoru Dohi, Shinji Kanemitsu, Hideto Shimpo, Masaaki Ito, Hajime Sakuma

**Affiliations:** 1 Department of Radiology, Mie University Hospital, Tsu-shi, Mie, Japan; 2 Department of Cardiology, Mie University Hospital, Tsu-shi, Mie, Japan; 3 Department of Thoracic and Cardiovascular surgery, Mie University Hospital, Tsu-shi, Mie, Japan

## Background

Stress myocardial perfusion MRI allows for accurate detection of flow-limiting stenosis in the coronary artery. However, reduced diagnostic accuracy of stress myocardial perfusion MRI was reported in patients with coronary artery bypass grafts (CABG). Flow measurement in CABG is another MR approach that permits functional assessment of graft stenosis. The purpose of this study was to evaluate the value of MR study that combines MR flow measurement of CABG and stress myocardial perfusion MRI in detecting graft stenosis.

## Methods

Thirty-two consecutive patients (26 men, 69.0 ± 7.0 years) with previous CABG surgery who had recurrent chest pain were studied. All patients gave informed consent and underwent both coronary angiography and cardiac MRI. After obtaining cine MRI, stress-rest myocardial perfusion MRI and late gadolinium enhanced (LGE) MRI, blood flow volume in the resting state was quantified in 61 graft conduits by using phase contrast cine MRI. The presence or absence of myocardial ischemia was visually determined by identifying stress-induced hypoenhancement in the absence of LGE or hypoenhancement larger than LGE. Stenoses ≥70% in grafts or grafted native vessels were considered significant on coronary angiography.

## Results

Coronary angiography revealed significant stenoses in the grafts or grafted native vessels in 19 (31%) of the 61 CABG conduits. Stress-rest perfusion CMR alone yielded the sensitivity and specificity of 73% and 81%, respectively. Receiver operating characteristic analysis demonstrated that the optimal cutoff values of MR blood flow volume was 28mL/min for predicting significant stenosis on angiography . By combining MR blood flow measurement and stress-rest perfusion MRI, the sensitivity and specificity of MR study were improved to 95% and 81%.

## Conclusions

MR flow measurement of CABG combined with stress-rest perfusion MRI can provide excellent diagnostic accuracy for predicting significant stenoses in the grafts or grafted native vessels in patients with previous CABG.

## Funding

Departmental reseach funding.

